# Motor Evoked Potentials after Supraspinal Stimulation in Pre- and Postoperative Evaluations of Patients with Cervical Radiculopathy

**DOI:** 10.1155/2019/4576493

**Published:** 2019-05-06

**Authors:** Aleksandra Bryndal, Magdalena Wojtysiak, Jakub Moskal, Joanna Lipiec-Kowalska, Martyna Borowczyk, Magdalena Tańska, Agnieszka Grochulska, Juliusz Huber, Marian Majchrzycki

**Affiliations:** ^1^Department of Physiotherapy, Pomeranian University, Slupsk, Poland; ^2^Department of Pathophysiology of Locomotor Organs, Poznan University of Medical Sciences, Poznan, Poland; ^3^Department of Neurosurgery and Neurotraumatology, Poznan University of Medical Sciences, Poznan, Poland; ^4^Department of Endocrinology, Metabolism and Internal Medicine, Poznan University of Medical Sciences, Poznan, Poland; ^5^Department of Rheumatology and Rehabilitation, Poznan University of Medical Sciences, Poznan, Poland

## Abstract

**Objective:**

Pre- and postoperative comparative evaluation of neurophysiological tests and clinical trials. Analysis of the diagnostic value of motor evoked potentials (MEP) induced by a magnetic field after supraspinal stimulation. Evaluation of the sensitivity and specificity of electromyography (EMG) and MEP is achieved.

**Methods:**

EMG, ENG, M-wave, F-wave, and MEP tests were performed on 35 patients with confirmed cervical radiculopathy in pre- and postoperative evaluations. The clinical trial consisted of evaluation of muscle strength, a sensory perception test and evaluation of tendon reflexes and pain severity.

**Results:**

The sensitivity of the resting EMG and MEP tests is 24%-67% and 6%-27%, while their specificity is 43%-80% and 86%-100%, respectively. The postoperative evaluation revealed a statistically significant reduction in pain severity (p=0001), an increase in muscle strength in DP (p=0.0431), BB (p=0,0431), and TB (p=0.0272), and improvement of touch sensation in terms of dermatomal innervation in C5 (p=0.0001) and C6 (p=0.0044).

**Conclusions:**

Tests comparing MRI sensitivity to neurophysiological tests show that neuroimaging is more sensitive in diagnostics of patients with cervical radiculopathy; however, clinical neurophysiology tests are more specific in reference to clinical trials.

## 1. Introduction

Pain in the cervical spine is a complex diagnostic and therapeutic problem occurring in various diseases [1, 2].

Neurophysiological tests are commonly used in the diagnosis of radiculopathy [3–6], but reports describing their limited diagnostic value are few [7]. In case of suspected cervical radiculopathy, electromyography (EMG), electroneurography (ENG) of the sensory (SCV), and motor fibers (M-wave) and F-wave tests [2–5] are used as the standard.

The use of motor evoked potentials (MEP) induced by a magnetic field is becoming more and more common [8, 9]. In neurophysiological diagnostics, the utility of magnetic supraspinal stimulation in patients with suspected radiculopathy is a subject of continuous study and discussion [10–13].

The aim of this study was to assess the effects of surgical treatment in the light of clinical and neurophysiological studies on a group of patients. This article also evaluates the diagnostic value of root-motor evoked potentials induced by the magnetic field after supraspinal stimulation (RxMEP).

## 2. Materials and Methods

The group of patients consisted of 35 people (16 women and 19 men) aged 31-68 (mean 54 ± 8 years), 155-188 cm tall (mean 168 ± 8 cm), with disc-root conflict confirmed by an magnetic resonance imaging (MRI) scan and/or lateral stenosis in the cervical spine. The patients had experienced pain for between 6 to 120 months (on average 43 ± 39 months) before the first examination, which had been severe for between 3 and 9 months (on average 6 ± 2 months). The patients were examined twice, before and after surgery. The time between the first examination and the surgery was 0.5-2 months (on average 1.0 ± 0.4 months), while that between the surgery and the second examination was 5-11 months (on average 6 ± 1 month).

The criteria for inclusion were as follows: symptoms of cervical spine radiculopathy noted in the clinical trial; disc-root conflict confirmed in an MRI, or lateral stenosis with compression of nerve roots in the cervical spine; neurosurgical qualification for surgical treatment; no contraindications for neurophysiological testing and no changes in the conduction of sensory fibres in upper limb nerves.

All patients were qualified by a neurosurgeon for cervical spine surgery. On 2 patients, a discectomy was performed at the C4/5 level; on 3 patients, at the C4/5 and C5/6 levels; on 7 patients, at the C5/6 level; on 11 patients, at the C5/6 and C6/7 levels; on 3 patients, at the C6/7 level and on 1 patient, a discectomy and interbody fusion were performed at the C4/5, C5/6, and C6/7 levels.

The clinical trial was conducted twice: before surgery on the day of the first neurophysiological test and after surgery on the day of the second neurophysiological test. The evaluation covered exteroceptive sensation, muscle strength based on the Lovett scale, and tendon reflexes and severity of pain (Visual Analogue Scale: VAS). For all patients, the MRI results were independently evaluated before surgery by a radiologist and a neurosurgeon. They determined the presence of spinal root compression as a result of disc-root conflict and/or lateral stenosis in the cervical spine, as well as the extent of the damage. Evaluation of root compression was verified intraoperatively by the surgeon. MRIs were performed before surgery (on average 2.9 ± 1.3 months).

**Figure 1:**
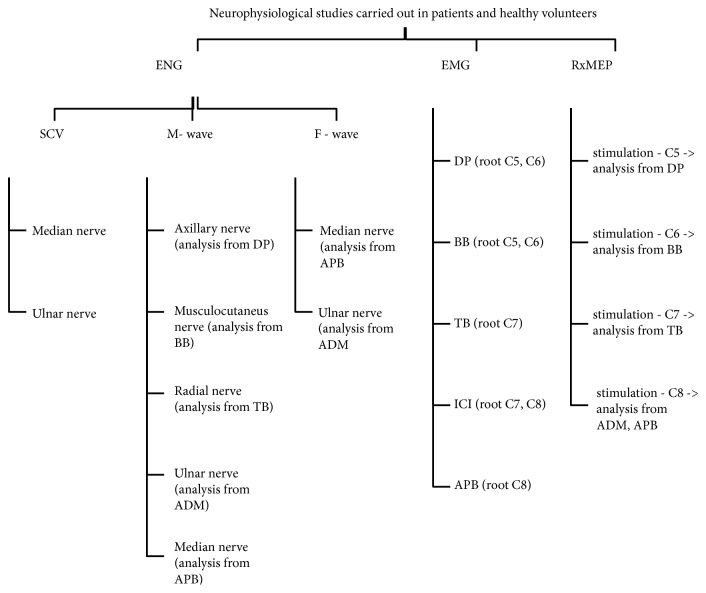
Diagram of the neurophysiological tests performed on the group of patients and the group of healthy volunteers. EMG: electromyography; ENG: electroneurography; SCV: sensory conduction velocity, RxMEP: root-motor evoked potentials induced by a magnetic field after supraspinal stimulation; ADM: abductor digiti minimi; APB: abductor pollicis brevis; BB: biceps brachii; DP: deltoid posterior; ICI: dorsal interosseus muscle I; TB: triceps brachii.

Figure 1 presents a set of applied neurophysiological tests in patients with C5-C8 ventral root damage. The needle electromyography was performed on at least two muscles of a given myotome but innervated peripherally by different nerves [8, 14]. Evaluated were the muscles of the upper limb on the affected side, namely, the deltoid posterior (DP, innervated from the C5 and C6 root level), the biceps brachii (BB, innervated from the C5 and C6 root level), the triceps brachii (TB, innervated from the C7 root level), the dorsal interosseus muscle I (ICI, innervated from the C7 and C8 root level), and the abductor pollicis brevis (APB, innervated from the C8 root level) [8, 14, 15].

An ENG test was used to evaluate nerve conduction in motor fibres of the axillary nerve and musculocutaneous nerve and in motor and sensory fibres of the radial nerve, median nerve, and ulnar nerve on the affected side. Also analysed in the first and second test were the parameters of the F-wave in motor fibres of the median and ulnar nerves.

The standard neurophysiological test was accompanied by an RxMEP test. The root response was recorded on both sides from the DP, BB, TB, and APB muscles, as well as the abductor digiti minimi (ADM) muscle. A C-100 circular coil, 110 mm in diameter, was used for stimulation. The stimulation was performed 3 times for each level tested by placing the coil centrally over the spinous process of a selected vertebra at the cervical level. A single magnetic impulse with a force equal to 100% of the stimulator ejection was used [13, 16]. The potentials with the highest possible amplitude and shortest latency were selected for the final analysis. The parameters of root responses were evaluated, including latency in milliseconds (ms) and amplitude in millivolts (mV). It was assumed that placement of the stimulation coil over the cervical spine would enable excitation of motor spinal roots where they exit the intervertebral foramina [9]. For this reason, F-wave conduction time (FCT) and root conduction time (RxCT) were calculated. FCT was calculated according to the following formula: (minimum F-wave latency + distal latency of induced response M – 1ms): 2 [11, 13, 17]. RxCT was calculated using the formula below: FCT: the shortest root-motor evoked potential latency (RxMEP latency) [11, 17]. The methodology of the RxMEP testing and the formulas used were described by Matsumoto et al. [13]. The FCT and RxCT parameters were calculated for the ICI and APB muscle recordings, as stimulation was performed and F-wave potential was recorded only for the median and ulnar nerves.

The test results from the group of patients were compared with their counterparts from the control group, which consisted of 76 healthy volunteers (44 women and 32 men) aged 22-72 (mean 46±13 years) and 150-195 cm tall (mean 171±10 cm).

Statistical analyses were performed using Statistica PL, version 10.0 (StatSoft, Inc., Cracow, Poland). Compliance of the analysed variables with the normal distribution was verified by the Shapiro-Wilk test (p>0.05). All the hypotheses considered in the study were verified at a significance level of p≤0.05 (marked in the tables with bold cells). Normative values were assumed (mean value ±2.5 SD).

To compare the results of the first and second trial, the Wilcoxon matched-pairs test was used (also, for associated variables, the t-Student test was used - these variables are marked in the tables with an asterisk).

To compare the test results of the patients with those of the control group, the Mann-Whitney test was used (also, for independent variables, the t-Student test was used - these variables are marked in the tables with an asterisk).

Spearman's rank correlation coefficient r_s_, that is, the correlation between the value of RxCT, RxMEP latency, and FCT, was calculated. The specificity and sensitivity of the respective neurophysiological tests were evaluated intraoperatively.

## 3. Results

The pre- and postoperative results of the clinical trials, MRIs, and neurophysiological tests for each patient are presented in Table 1.

Recorded in the clinical trial after surgery were a statistically significant reduction (p=0001) in pain severity measured using the VAS scale, an increase in muscle strength measured using the Lovett scale in DP (p=0.0431), BB (p=0,0431), and TB (p=0.0272), and improvement of touch sensation with respect to the dermatomal innervation of C5 (p=0.0001) and C6 (p=0.0044). No statistically significant improvement in the normalization of tendon reflexes from TB, BB, or BR muscles was recorded.

**Figure 2:**
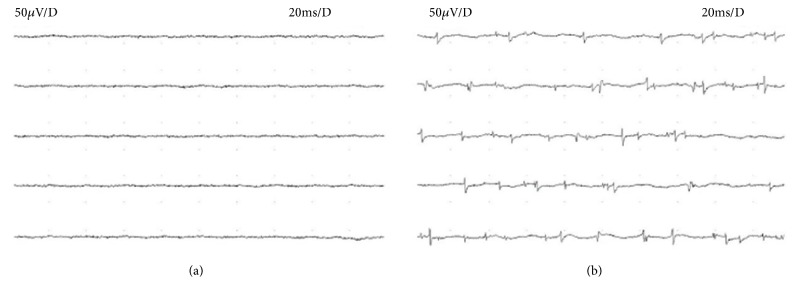
Resting records from basic EMG performed on the abductor pollicis brevis muscle in (a) a healthy volunteer from the control group, and in (b) a patient with cervical radiculopathy. Part (a) of the figure shows “electric silence,” i.e., the lack of pathological spontaneous potentials, while part (b) shows positive sharp waves and fibrillations.

**Figure 3:**
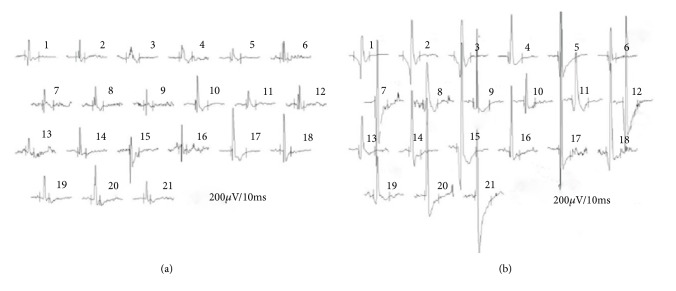
MUAP recordings from the basic EMG on the abductor pollicis brevis muscle in (a) a healthy volunteer from the control group, and in (b) a patient with cervical radiculopathy. Part (a) of the figure shows the MUAP with correct parameters for amplitude, duration, and area, and part (b) of the figure shows the MUAP with increased values of the parameters for amplitude, duration and area.

When analysing the parameters of the needle electromyography during rest in the preoperative period, resting denervation activity was observed (Figure 2(b)) in 30 patients in the following muscles: DP n=9, BB n=10, TB n=7, APB n=13, and ICI n=15 (dorsal interosseus muscle I). In the remaining patients, electrical silence was observed (Figure 2(a)). When testing motor action unit potentials (MUAP) under conditions of voluntary contraction, reinnervation (Figure 3(b)) was observed in 24 patients in the following muscles: DP n=7, BB n=8, TB n=15, APB n=9, and ICI n=13. In the remaining patients, the MUAP parameters were correct (Figure 3(a)). During the postoperative period, resting denervation activity was recorded in only 7 patients in the following muscles: DP n=1, BB n=1, TB n=1, APB n=3, and ICI n=3. However, the reinnervation process was recorded in 24 patients in muscles DP n=10, BB n=11, TB n=16, APB n=10, and ICI n=13 during the MUAP test under voluntary contraction conditions.

In the ENG test, axonal changes were observed only in one patient. They were expressed by a decrease in M-wave amplitude during the first and second trial in axillary nerve motor conduction in DP (MRI – C5, C6 root damage level).

In the ENG test of the M-wave in motor fibres of the median and ulnar nerves during the first and second trial, elongation of F-wave latency was observed in two patients. F-wave frequency reduction was demonstrated in three patients during the first trial, while an improvement was observed in two persons during the second trial (MRI – C6, C7 root damage level).

The FCT parameter was observed to be prolonged in two patients after stimulation of the median nerve during the first trial and in one these two during the second trial. In these patients, the MRI scan showed damage to the C6 and C7 roots, as well as prolongation of the minimum F-wave latency after median nerve stimulation during the first and second trial. In one of these persons, prolongation of the FCT was recorded during the first trial, as well as after ulnar nerve stimulation. The minimum F-wave latency was also prolonged during the first and second trial. During the second trial, FCT was within the normal range.

A closer analysis of RxMEP at the level of C5 and the recording from DP revealed latency prolongation in 4 patients during both the first and second trial. The RxMEP latency parameter during the recording from APB was prolonged in only one patient during the pre- and postoperative period.

Prolongation of the RxCT parameter was observed in only one patient when recording the response from ADM and APB muscles at two stages. However, in one patient, prolongation of the RxCT parameter in the ADM muscle was observed at two stages. The observations mentioned above suggest that the RxCT parameter has no significant value in the diagnostics and evaluation of patients with cervical radiculopathy, which is shown by the data in Table 2.

Table 3 presents the results of correlation for selected RxMEP and F-wave tests (RxCT, RxMEP latency, and FCT) in APB and ADM muscles during the first trial, without division by root damage level. No statistically significant correlation was found between RxCT and RxMEP latency, or between RxCT and FCT for both tested muscles. Based on the aforementioned analyses, it can be concluded that the RxCT parameter is of no diagnostic significance in patients with cervical radiculopathy. Therefore, in the neurophysiological diagnostics of cervical radiculopathy, the evaluation of conduction in the spinal root motor fibres must be based on the RxMEP latency parameter.

**Figure 4:**
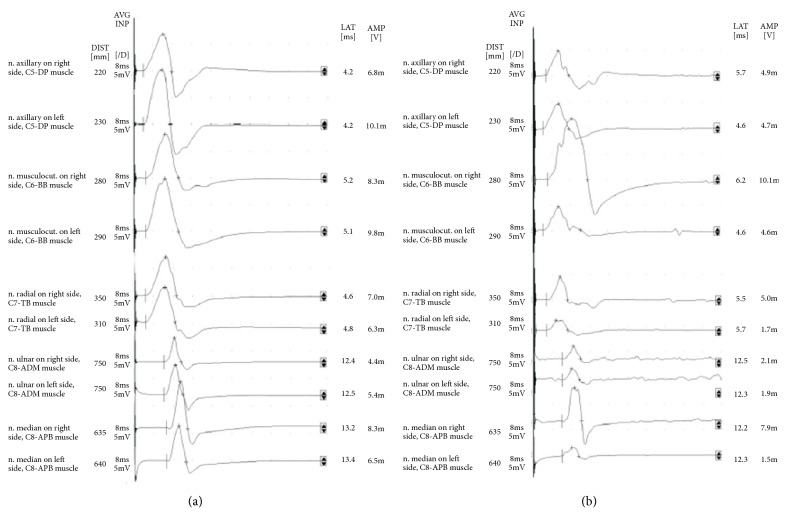
Recordings of root-motor evoked potentials (MEP) induced by a magnetic field, performed bilaterally (a) in a healthy volunteer and (b) in a patient with disc-root conflict. What must be noted in the case of the patient is the asymmetry of responses induced from the same muscles on the right and left sides, expressed by a reduction in amplitude or a prolonged latency parameter.

Table 4 comprises the results of the RxMEP and F-wave tests, with a division of patients by root damage found in the MRI at levels C5, C6, C7, and C8. The amplitude parameter (Figure 4) turned out to be diagnostically significant based on statistical analyses of both damage to spinal roots C5, C6, and C7, as well as this conduction in the postoperative analysis. This is contrary to general statements that root response amplitude is of no diagnostic significance due to its great variability, and the impossibility to apply a magnetic incentive, which would be a supramaximal incentive. Prolongation of root response latency turned out to be diagnostically significant only for C6 spinal nerve damage.

The EMG test of the BB muscle shows the highest sensitivity and specificity for both C5 and C6 root damage. In the case of C7 root damage, the sensitivity disclosed as a result of electromyography of the ADB muscle is higher than that of the TB muscle; however, electromyography of the TB muscle shows higher specificity than electromyography of the ADB muscle. The RxMEP test is characterised by very high specificity in detection of C5-C7 root damage and a relatively low diagnostic sensitivity ranging between 6 and 27% (Table 5).

## 4. Discussion

Basic EMG revealed neurogenic changes in 96% of the patients. These changes matched the level of damage to a given spinal root confirmed in the MRI scan and intraoperative evaluation. These results are consistent with the studies of other authors who determined the sensitivity of this test at 94-98% when analysing EMGs of 6 muscles [4, 18]. Yet an overview of 9 papers conducted by AANEM (1999) [18] in reference to the diagnostics of patients with cervical radiculopathy indicates the sensitivity of needle EMG as being between 50 and 71%. In studies performed by Leblhuber et al. [19] changes were observed in needle EMG in 60% of the patients with cervical radiculopathy. In this research, the EMG sensitivity to determine the C5-C8 damage level ranged between 67 and 24% and, for the MEP test, between 6 and 27%.

Hakimi and Spanier [2] claim that needle electromyography is the most useful test in radiculopathy diagnostics, as it can localise changes at a specific root level, provide information on the stage at which the radiculopathy is diagnosed (initial, development, and chronic) [20], and indicate whether the axons of motor fibres are damaged [15]. However, it is necessary to remember the limitations of this test, as many radiculopathies at an early development stage may be characterised only by changes in the sensory fibres of the spinal roots, or only by demyelinating changes [2]. The result of the EMG may be correct even if radiculopathy is the cause of severe pain [19, 21]. In this paper, a false positive of the basic EMG was noted only for the C7 root in 19% of patients. Studies conducted by Ashkan et al. [7] suggest that clinical evaluation of patients with cervical radiculopathy should be based mainly on MRIs, whose sensitivity they determined at 93%. On the other hand, neurophysiological tests whose sensitivity was determined at 42% are not necessary for routine diagnostics.

The ENG test of motor fibres is a low-sensitivity method in the evaluation of cervical radiculopathy. This is because in most radiculopathies, only a small percentage of motor fibres in ventral roots is damaged. Only if loss exceeds 50% can the evoked response amplitude be reduced when evaluating muscles supplied by fibres coming from the damaged root in comparison to the opposite side not affected by pathology [19].

Analysis of the results of F-wave tests from the median and ulnar nerves during the pre-operative period showed irregularities in only 22% of the patients with cervical radiculopathy, confirmed by MRIs at C7 and C8. These results are consistent with studies conducted by Leblhuber et al. (1988) [20]. In 8% of patients, prolonged F-wave latency was observed, while in 19%, reduced F-wave frequency was observed. The low percentage of irregularities in this test may be a consequence of the small number of patients with cervical radiculopathy among those with damage at C7 and C8. Lo et al. [22] as well as Hakimi and Spanier [2] point out that, for this disease, the F-wave test result is not sufficiently sensitive but may demonstrate irregularities at a late period of the untreated disease and in case of severe root damage. By means of the F-wave test, it is possible to isolate the damage of only one root due to the innervation of several root levels of muscles from which this response is recorded [2, 22]. Lin et al. [23] carried out a detailed analysis of patients with cervical radiculopathy confirmed by an MRI at the C7 and C8 roots. They obtained F-wave recordings after a simulation of the median and ulnar nerves. They considered the sensitivity and specificity of this test to be low (sensitivity: 0.09-0.52). Studies conducted by other authors determine the sensitivity of this test to be at a slightly higher level, i.e., 10-20% [24].

Neurophysiological tests are perceived by many authors as helpful in the diagnostics of patients with cervical radiculopathy. Needle electromyography seems to be a precise and commonly accepted method among all electrodiagnostic procedures followed in radiculopathy diagnostics [6, 25, 26]. Knutsson [27] reports that needle electromyography results are correlated with intraoperative evaluation of damage to spinal nerve roots in 79% of patients. He based this observation on the presence of fibrillation and positive sharp waves during EMG tests in limb muscles on the affected side. Patients with cervical radiculopathy in whom positive sharp waves and fibrillation were detected via needle EMG had better results in the postoperative evaluation than those in whom no changes were observed in the EMG [28]. The evaluation of sensitivity and specificity in this paper is based on the presence of fibrillation and positive sharp waves in the upper limb muscles on the affected side.

Ugawa et al. [16] observed that magnetic stimulation with a circular coil situated over the spinous process of a selected cervical vertebra might excite spinal nerves in the intervertebral foramen. The latency parameters during magnetic excitation and electrical excitation at the level of the spine are almost identical. Root response latency is always shorter than FCT, which is calculated using F-wave parameters. It is therefore argued that as a result of magnetic stimulation in the spine, the excitation of spinal nerves takes place at the level of the intervertebral foramen [13]. Other authors confirm clinical application of this method [16, 29] using a circular coil [16, 30] or an 8-shape coil [31]. Potential latency after stimulation by the magnetic field at the level of the intervertebral foramen is a parameter which reflects the correctness of the peripheral conduction of nerve impulses from the intervertebral foramen to the muscle from which the response is recorded. This parameter does not reflect the conduction of nerve impulses at the level of ventral roots in the spinal canal, or/i.e., from the level of the motor cell body to the intervertebral foramen of the cervical spine. This is why RxCT was calculated. According to Hallet and Chokroverta [9], the mean RxCT for spinal roots is 1.4ms. In this research, the mean RxCT ranged between 0.7 and 0.9 ms. This time is considered to be too short for precise evaluation of root conduction; therefore, it is usually not subject to analysis [13]. In our research, it also turned out to be a parameter of no diagnostic significance for evaluation of the extent and level of damage to spinal nerves. Changes in this parameter were observed in only 7% of the patients in the pre- and postoperative evaluations, as compared to the results obtained in the control group. RxCT, however, is a significant parameter in diagnostics of root damage in the lumbosacral spine, which has been confirmed by the studies of Wojtysiak et al. [32]. It is worth noting that the root response latency parameter not only reflects peripheral nerve conduction but also includes delay time on the nerve-muscle synapse and the depolarisation time necessary to generate muscle action potentials [13, 16]. For this research, an RxMEP test was performed on all patients, and irregularities were found in 19%. Hence the conclusion that the RxMEP test is much less sensitive than needle EMG in the evaluation of damage to cervical roots (Table 5). This claim is consistent with studies conducted by Menkesa [12]. However, there is no doubt that the use of several diagnostic methods allows for an increase in the sensitivity of neurophysiological tests on patients with cervical spondylosis. This enables confirmation of purely demyelinating changes in the motor fibres of spinal roots, which are diagnostically silent in EMG. A parameter that turned out to be significant in pre- and postoperative diagnostics was RxMEP amplitude, which was subject to a statistically significant increase in patients after operative treatment of cervical radiculopathy. Therefore, the RxMEP test may serve as an objective diagnostic tool for patients after operative treatment.

Clinical neurophysiology and neuroimaging focus on different aspects of spinal root damage. Neurophysiological tests detect functional pathology, while neuroimaging detect structural pathology. Each of the these tests is characterised by different benefits and limitations. Tests comparing MRI sensitivity to neurophysiological tests show that neuroimaging is more sensitive in diagnostics of patients with cervical radiculopathy; however, clinical neurophysiology tests are more specific in reference to clinical trials. It is therefore believed that these two diagnostic methods may be highly complementary. Neurophysiological tests should be implemented when there is a discrepancy between the results of MRIs and the clinical trials [33].

## 5. Conclusions

1. Surgical removal of the cause of compression on the spinal nerve root in the cervical spine significantly improves the clinical condition of patients.
2. Among the neurophysiological methods used in this study, basic EMG turned out to be the most important to evaluation of nerve root damage in the cervical spine.
3. Root-motor potentials are an important diagnostic test in the evaluation of surgical treatment.
4. Root conduction time (RxCT) is of little diagnostic significance to evaluation of patients with cervical spondylosis.
5. This research has indicated that electroneurographic tests of motor fibres (M-wave and F-wave) are complementary to the diagnostics of cervical radiculopathy.

## Figures and Tables

**Table 1:** Pre- and post-operative clinical trials, MRI scan and neurophysiological tests on 35 patients with root conflict in the cervical spine.

Patient number	Sex	MRI	Operation	Clinical trial								Neurophysiological test			
		MRI – level of damage to spinal root	Operation level	Lovett scale (motor deficit)		Sensation deficits		Reflexes		VAS		EMG		MEP	
				Before	After	Before	After	Before	After	Before	After	Before	After	Before	After
1	F	C5, C6, C7	C4/5, C5/6	N	N	N	N	N	N	3	1	N	N	N	N
2	F	C6, C7	C5/6, C6/7	N	N	C5, C6	N	C5-C6↓	N	4	2	C5, C6	N	N	N
3	F	C6	C5/6	N	N	N	N	N	N	4	3	C7, C8	N	N	N
4	F	C6	C5/6	C7, C8	C8	N	N	N	N	5	3	N	N	N	N
5	F	C5	C4/5	C5, C6, C7	N	N	N	N	N	4	2	C8	N	N	N
6	F	C7	C6/7	N	N	N	N	C5-C6↓	C5-C6↓	6	4	N	N	N	N
7	F	C6, C7, C8	C5/6, C6/7	C8	N	C6, C7	N	C5-C6↓	C5-C6↓	1	1	C7, C8	C8	N	N
8	F	C7	C6/7	C8	C8	N	N	N	N	4	4	C8	N	N	N
9	F	C6, C7	C5/6, C6/7	C5, C6	N	N	N	C5-C6↓	N	6	3	C5	N	N	N
10	F	C4, C5, C6, C7	C5/6	C5	N	N	N	C5-C6↓	C5-C6↓	5	3	C5	N	N	N
11	F	C4, C6, C7	C5/6, C6/7	N	N	N	N	C5-C6↓, C7↓	C5-C6↓, C7↓	2	1	C5, C6, C7	N	N	N
12	F	C6	C5/6	N	N	N	N	C5-C6↓	C5-C6↓	5	3	N	N	N	N
13	F	C6	C5/6	C6	C6	C6	N	C5-C6↓, C7↓	C5-C6↓, C7↓	6	5	C6, C7	N	N	N
14	F	C5	C4/5	N	N	C6, C7, C8	C6, C7, C8	C5-C6↓	N	6	2	C5, C6, C7, C8	C7, C8	N	N
15	F	C6, C7	C5/6, C6/7	N	N	N	N	C5-C6↓	C5-C6↓	5	3	C5, C6	N	N	N
16	F	C6	C5/6	N	N	C7, C8	C7, C8	N	N	3	2	C8	N	N	N
17	M	C7, C8	C6/7	N	N	C7, C8	C7, C8	C5-C6↓	C5-C6↓	4	5	C8	N	N	N
18	M	C6, C7, C8	C5/6, C6/7	C5, C6, C7, C8	C5, C8	C7, C8	N	N	N	5	7	C8	N	N	N
19	M	C4, C5, C6, C7, C8	C4/5, C5/6, C6/7	N	N	N	N	C5-C6↓	N	1	1	C6, C8	N	C8	C8
20	M	C5, C6, C7	C4/5, C5/6, C6/7	C5, C6	C5, C6	N	N	C5-C6↓, C7↓	C5-C6↓	4	0	C5, C6, C7	N	N	N
21	M	C4, C5, C6	C4/5, C5/6	C8	N	C5, C6	N	C5-C6↓	C5-C6↓	6	4	C5, C6	N	C8	C8
22	M	C6, C7	C5/6, C6/7	C5, C6, C7, C8	C5, C6, C7, C8	N	N	N	N	6	5	C8	C8	C8	C8
23	M	C7	C6/7	N	N	C7, C8	C7, C8	C5-C6↓	C5-C6↓	4	5	C8	N	N	N
24	M	C4, C6, C7	C5/6, C6/7	N	N	C5, C6	N	C5-C6↓	N	8	6	C5, C6, C7, C8	C5, C6	C8	C8
25	M	C7, C8	C5/6, C6/7	C5, C6, C7, C8	C5, C6, C7, C8	N	N	N	N	6	5	C8	C8	C8	C8
26	M	C6, C7	C5/6, C6/7	N	N	C7, C8	C7, C8	C5-C6↓, C7↓	C5-C6↓, C7↓	4	2	C8	N	C8	C8
27	M	C6, C7	C5/6, C6/7	C5, C6, C7, C8	C5, C8	C7, C8	N	N	N	5	7	C8	N	N	N
28	M	C5, C6, C7	C5/6, C6/7	C5, C6, C7	C5, C6, C7	N	N	C5-C6↓, C7↓	C5-C6↓, C7↓	7	4	N	N	N	N
29	M	C6, C7	C5/6, C6/7	C5, C6, C7	N	C5, C6	N	C5-C6↓	C5-C6↓	7	4	C6	N	N	N
30	M	C4, C6, C7, C8	C5/6, C6/7	N	N	C5, C6	N	C5-C6↓	N	8	6	C5, C6, C7, C8	C5, C6	C8	C8
31	M	C6, C7, C8	C5/6, C6/7	N	N	C7, C8	C7, C8	C5-C6↓, C7↓	C5-C6↓, C7↓	4	2	C8	N	C8	C8
32	M	C4, C5, C6, C7	C4/5, C5/6, C6/7	N	N	N	N	C5-C6↓	N	1	1	C6, C8	N	C8	C8
33	M	C4, C6	C5/6	N	N	N	N	C5-C6↓	C5-C6↓	4	2	C8	N	N	N
34	M	C6, C7, C8	C5/6, C6/7	C6, C7, C8	C6, C7, C8	N	N	C7↓	C7↓	4	0	C6, C7, C8	N	N	N
35	M	C7, C8	C5/6, C6/7	C7, C8	N	C7	N	N	N	6	4	C6, C7	N	C8	C8

Total	K-16 M-19	C5 – 9 C6 – 27 C7 – 26 C8 – 9	C5 – 7 C6 – 29 C7 – 24 C8 – 0	C5 – 10 C6 – 11 C7 –10 C8 – 9 N - 18	C5 – 6 C6 – 6 C7 – 4 C8 – 5 N - 25	C5 – 5 C6 – 8 C7 – 10 C8 – 7 N - 19	C5 – 0 C6 – 1 C7 – 6 C8 – 6 N - 29	C5-C6↓ – 23 C7↓ – 7 N – 11	C5-C6↓ – 16 C7↓ – 9 N – 18	Median (range) 5 (1-8)	Median (range) 3 (0-7)	C5 – 10 C6 – 14 C7 – 10 C8 – 20 N - 5	C5 – 2 C6 – 2 C7 – 1 C8 – 4 N - 29	C5 – 0 C6 – 0 C7 – 0 C8 – 10 N - 25	C5 – 0 C6 – 0 C7 – 0 C8 – 10 N - 25

Before – before surgery; After – after surgery; Reflexes: C5-C6 - biceps, brachioradialis muscle, C7 - triceps; ↓ - weakening of the tendon reflex; N- normal

**Table 2:** Results of the Wilcoxon matched-pairs test (the t-Student test was also used for associated variables, marked in the tables with an asterisk), which show significant differences (bold field) between individual values of parameters that determine root conduction in the pre- and postoperative evaluations.

Spinal root level (N=27)	Lat. Min. F	Freq. F	FCT	RxMEP latency	RxMEP amplitude	RxCT	M-wave amplitude
C5 (recording from DP	nr	nr	na	*∗*0.0786	**0.0044**	nr	*∗*0.2333

C6 (recording from BB)	nr	nr	na	*∗ * **0.0035**	*∗*0.5521	nr	*∗*0.6343

C7 (recording from TB)	nr	nr	na	*∗*0.1437	*∗*0.1882	nr	*∗*0.2149

C8 (recording from TB)	0.6938	0.6164	*∗*0.5247	*∗*0.2665	*∗ * **0.0335**	0.3305	0.7032

C8 (recording from ADM)	*∗ * **0.0098**	0.1424	*∗ * **0.0306**	*∗*0.6656	*∗*0.5564	0.1183	*∗*0.2577

F min. APB: minimum F-wave latency after stimulation of motor fibres, Freq. F: frequency of F-waves after stimulation of nerve motor fibres, FCT: F-wave conduction time, RxMEP: root motor evoked potentials, RxCT: root conduction time, DP: deltoid posterior, BB: biceps brachii, TB: triceps brachii, APB: abductor pollicis brevis, ADM: abductor digiti minimi, nr: nonrecorded, and na: nonanalyzed.

**Table 3:** Correlation (Spearman's rank correlation coefficient rs) between root conduction time parameter (RxCT), RxMEP latency, and F conduction time (FCT). Recordings from the MEP abductor pollicis brevis muscle (APB) and abductor digiti minimi muscle (ADM) were compared.

	N	muscle	r_s_	p
RxCT vs. RxMEP latency	35	APB	-0.17	0.4032
	35	ADM	-0.23	0.2468

RxCT vs FCT	35	APB	0.33	0.0898
	35	ADM	0.32	0.1008

**Table 4:** Results of the Wilcoxon matched-pairs test, which show significant differences (bold field) between the individual values of parameters determining root conduction in the pre- and postoperative evaluations, with groups divided by spinal root damage revealed in the MRI scan.

Spinal root damage	Amp. MEP DP	Lat. MEP DP	Amp. MEP BB	Lat. MEP BB	Amp. MEP TB	Lat. MEP TB	Amp.MEP APB	Lat. MEP APB	F min. APB	Freq. F APB	FCT APB	RxCT APB
C5 (n=9)	**0.0117**	0.2936	**0.0117**	0.1508	nr	nr	nr	nr	nr	nr	nr	nr
C6 (n=27)	**0.0251**	**0.0496**	0.0796	**0.0011**	nr	nr	nr	nr	nr	nr	nr	nr
C7 (n=26)	nr	nr	nr	nr	0.1329	0.0582	**0.0209**	0.3719	0.4459	0.9645	0.2575	0.2959

C8 (n=9)	nr	nr	nr	nr	0.2421	0.0671	0.0612	0.0968	0.2439	0.0724	0.0695	0.4112

Amp. MEP: MEP amplitude after supraspinal magnetic stimulation, Lat. MEP: motor evoked potential latency after supraspinal magnetic stimulation, F min. APB: minimum F-wave latency after stimulation of the median nerve motor fibres recorded from the abductor pollicis brevis muscle, Freq. F APB: F-wave frequency after stimulation of the median nerve motor fibres and recording from the abductor pollicis brevis muscle, FCT APB: F conduction time after stimulation of motor fibres of the median nerve and recording from the abductor pollicis brevis, RxCT APB: root conduction time for motor fibres of the median nerve, DP: deltoid posterior, BB: biceps brachii, TB: triceps brachii, and nr: nonrecorded

**Table 5:** Sensitivity and specificity of clinical neurophysiology tests in the evaluation of patients undergoing surgery for radiculopathy of spinal roots C5, C6, and C7.

	Sensitivity (%)	Specificity (%)
EMG recording from DP (C5 decompression)	43	75
EMG recording from DP (C6 decompression)	36	80
EMG recording from BB (C5 decompression)	67	76
EMG recording from BB (C6 decompression)	41	80
EMG recording from TB (C7 decompression)	24	70
EMG recording from APB (C7 decompression)	33	43
RxMEP recording from DP (C5 decompression)	20	86
RxMEP recording from DP (C6 decompression)	27	100
RxMEP recording from BB (C5 decompression)	17	90
RxMEP recording from BB (C6 decompression)	14	100
RxMEP recording from TB (C7 decompression)	6	100
RxMEP recording from APB (C7 decompression)	13	100

EMG: electromyography, RxMEP: root motor evoked potentials, DP: deltoid posterior, BB: biceps brachii, TB: triceps brachii, and APB: abductor pollicis brevis.

## Data Availability

The [MRI results, neurophysiological examination, and clinical examination of patients] data used to support the findings of this study are available from the corresponding author upon request.
